# Validation of French versions of the 15-item picker patient experience questionnaire for adults, teenagers, and children inpatients

**DOI:** 10.3389/fpubh.2024.1297769

**Published:** 2024-02-19

**Authors:** Clement P. Buclin, Adriana Uribe, Justine E. Daverio, Arnaud Iseli, Johan N. Siebert, Guy Haller, Stéphane Cullati, Delphine S. Courvoisier

**Affiliations:** ^1^Division of Quality of Care, University Hospitals of Geneva, Geneva, Switzerland; ^2^Department of Medicine, University of Geneva, Geneva, Switzerland; ^3^Department of Sociology, Geneva School of Social Sciences, University of Geneva, Geneva, Switzerland; ^4^Division of Paediatric Emergency Medicine, Department of Women, Child and Adolescent, University Hospitals of Geneva, Geneva, Switzerland; ^5^Population Health Laboratory (#PopHealthLab), Faculty of Science and Medicine, University of Fribourg, Fribourg, Switzerland

**Keywords:** patient satisfaction, hospitals, healthcare quality, healthcare access, healthcare evaluation, validation study, inpatients background

## Abstract

**Objectives:**

No French validated concise scales are available for measuring the experience of inpatients in pediatrics. This study aims to adapt the adult PPE-15 to a pediatric population, and translating it in French, as well as to establish reference values for adults, teenagers, and parents of young children.

**Methods:**

Cultural adaptation involved forward and backward translations, along with pretests in all three populations. Dimensional structure and internal consistency were assessed using principal component analysis, exploratory factor analysis, and Cronbach's alpha. Construct validity was assessed by examining established associations between patient satisfaction and inpatient variables, including length of stay, and preventable readmission.

**Results:**

A total of 25,626 adults, 293 teenagers and 1,640 parents of young children completed the French questionnaires. Factor analysis supported a single dimension (Cronbach's alpha: adults: 0.85, teenagers: 0.82, parents: 0.80). Construct validity showed the expected pattern of association, with dissatisfaction correlating with patient- and stay-related factors, notably length of stay, and readmission.

**Conclusion:**

The French versions of the PPE-15 for adults, teenagers and parents of pediatric patients stand as valid and reliable instruments for gauging patient satisfaction regarding their hospital stay after discharge.

## Background

In recent years, there has been an increasing emphasis on patients' satisfaction, widely acknowledged as an indicator for quality of care improvement (1–3). This aims at enhancing the quality of care in hospitals (4). Higher satisfaction of patients with the care provided has demonstrated associations with improved adherence (5), continuity (6) and intercontinuity of care (7), reduced care utilization (8), as well as more efficient care (9). Furthermore, patients' experience measures are excellent tools to benchmark hospitals and guide the development of programs aimed at enhancing patient satisfaction (10). They also provide a rapid and low-cost feedback to clinicians on the care provided (11). In response to these findings, a growing number of countries have included satisfaction measurement into their quality improvement processes (12). In the UK for instance all National Health Service (NHS) centers are assessing patient experience since 2002 through the NHS Patient Survey Program (13). Switzerland introduced nationwide surveys in acute care hospitals in 2009 and in rehabilitation clinics from 2013 (10).

Most surveys in these countries and others are based on self-developed questionnaires that often differ between or even within hospitals and other healthcare organizations (14–16). This has several pitfalls. One is the absence of homogeneity of measurement tools resulting in unreliable benchmarks between hospitals. Another is the additional complexity for quality improvement teams to implement programs that are homogeneous and easy to implement within institutions. Furthermore, those homegrown satisfaction surveys often contain many questions and longer surveys have been shown to decrease both precision and participation (17). Therefore, there is a need for standardized short tools that possess robust psychometric attributes, especially in terms of validity and reliability.

In the pursuit of standardizing and continuously enhancing the quality of care, numerous organizations have developed and validated questionnaires that target various aspects of patients' satisfaction with care, commonly referred to as Patient-Reported Experiences Measures. These questionnaires serve diverse purposes, with some explicitly evaluating patient satisfaction, such as the Patient Satisfaction Questionnaire-18 (PSQ-18) or the Press Ganey, while others, like the Hospital Consumer Assessment of Healthcare Providers and Systems (HCAPS) or the Picker Patient Experience Questionnaire, are more focused on patient experience (18–21). Patient experience differs from patient satisfaction; the former extends beyond the assessment of care quality to encompass a broader evaluation of patients' interactions with the healthcare system (22).

Over the past two decades, the Picker Institute has engaged in producing satisfaction questionnaires for inpatients that have robust psychometric properties (23). In 2002, the Institute developed the ≪ Picker Patient Experience Questionnaire 15 ≫ (PPE-15), a short set of 15 items validated for assessing the satisfaction of adult inpatients with their hospital stay after discharge (21). Its qualities and the fact that it explores seven different dimensions of patient care has led to its extensive use and translation in many non-English-speaking countries (24, 25).

However, there is currently no validated French translation of the PPE-15. Moreover, the PPE-15 primarily focuses on gauging satisfaction among adult inpatients, leaving a gap in concise instruments for evaluating the satisfaction of hospitalized children, their parents, and teenage inpatients. Due to its historical and recent popularity and its validation in several languages, developing and validating a French version of the PPE-15 adapted for all patients seen in our institution would not only allow a more extensive evaluation of patient satisfaction but also lead to benchmarking opportunities with the growing number of institutions using culturally adapted PPE-15.

The objectives of this study are threefold: (I) to adapt the adult PPE-15 for parents of young children inpatients and teenage inpatients, (II) to translate the adult PPE-15, as well as the newly adapted versions for pediatrics, into French, and (III) to establish reference values and conduct an initial comparative analysis of satisfaction across all three population groups.

## Methods

### Population

The University Hospitals of Geneva serves as a tertiary, academic, acute-care hospital with a capacity of over 2000 beds, encompassing a wide spectrum of medical specialties.

This study included three distinct inpatient populations. First, adult participants comprised patients aged over 16 years with a valid email or mobile phone number. The collection of email addresses or mobile phone numbers took place during hospital admission and was recorded by administrative clerks. Second, pediatric patients were categorized into two groups based on their age at discharge: patients older than 12 years received the teenager questionnaire, while patients 12 years or younger were administered the children's questionnaire, which was distributed to their parents.

Patients were contacted within a timeframe of 10 to 17 days following the discharge from the University Hospitals of Geneva. This period was selected to allow patients to address questions pertaining to the dimensions of care continuity and transition. Surveys were administered via a REDCap electronic data capture tool form hosted at the Geneva University Hospital (26, 27). The form was distributed to all discharged patients between October 2019 and December 2019, as well as from March 2020 to April 2023, with the aim of reaching an average of ~500 patients per week. Exclusion criteria included a hospital stay of < 24 h, absence of a cellphone number or email, and having already received a satisfaction survey in the last 3 months. As part of the process, up to two automated reminders were dispatched at 6-day intervals.

### Cultural adaptation, and translation of the pediatric PPE-15

The process of culturally adapting and translating the pediatric PPE-15 involved a pilot procedure led by a group of experts, all of whom were native French speakers. The team consisted of a pediatric nurse, a pediatric emergency doctor, a health sociologist, a psychologist, and a parent of a young child. The procedure comprised two steps:

#### Selection of survey items for patient satisfaction assessment among teenagers and parents of children

The group of experts adopted a sequential approach in selecting items. First, as the adult English version of the PPE-15 already existed and included chosen items from the long Picker Patient Experience questionnaires, experts extended this selection to include items from the Children long PPE questionnaires. This step aimed to comprehensively assess all dimensions of patient satisfaction outlined in the PPE-15 across both adult and pediatric age categories. Subsequently, the experts, notably the pediatrician and the parent of a patient, identified a dimension of patient satisfaction specific to the pediatric population: parental involvement. To address this dimension, two additional items assessing parental involvement were selected from the long questionnaire for the pediatric population. Special attention was given to adapting the language for questions aimed at teenagers to directly engage with them, addressing them instead of their parents.

#### Scale translation

Two proficient translators with expertise in the healthcare sector independently conducted the translation for all three questionnaires from English to French (forward translation). Subsequently, the team of experts scrutinized these translations, opting for those that most accurately captured the intended essence of the questions, leading to the creation of a unified version. Following this, two native English translators independently rendered this French version back into English (backward translation). The translators performing the backward translation were not privy to the original English questionnaire. The team of experts reviewed and validated the resulting English translations, and in some, adjustments were made to realign the questions with their initial intended meaning.

### Pre-test

The French versions of the PPE-15 underwent a pre-testing phase involving individual interviews conducted by three interviewers: a research nurse, a medical student, and a psychologist. These interviews were carried out at the University Hospitals of Geneva and involved voluntary inpatients arbitrarily chosen as representatives of the respective targeted populations. For the adult PPE-15, the pre-tests took place in rehabilitation, medicine, and surgery wards. As for the children and the teenagers' PPE-15, the pre-tests were held within various divisions of the pediatric department, including general medicine, surgery, orthopedics, neonatology, developmental medicine, haemato-oncology, and pediatric psychiatry. The aims of these interviews were twofold: to ensure that the translated items were clear and comprehensible across diverse inpatients populations, and to assess content validity by considering patient perspectives. After accepting to participate, patients had to answer the questionnaires as if in real conditions without interruptions. An investigator then elicited, for each item, any difficulties answering it. At the end, they were also asked about any item lacking to fully express their experience with their hospitalization. Feedbacks were obtained in 5 sessions for adults, and 7 for parents and adolescents. After each session, the questions were examined and modified if necessary, so that the next session would present the new formulation of the questions.

### Measurements and construct validity

Collected variables were age, gender, nationality, self-reported primary language, length of hospital stay (LOS), hospital departments of stay, number of positive and negative comments left in the satisfaction questionnaires and preventable readmissions. Comments left by patients in an optional box of the questionnaires are coded as positive or negative in the normal workflow of the patient satisfaction team of the University Hospitals of Geneva. One comment could be flagged several times to account for several positive or negative complaints in the text left by the patient. For hospital departments, while the category of stay in the psychiatric department was expected to have a low number of cases compared for instance to surgery, its potential association with satisfaction warranted its collection and inclusion in our analyses. Preventable readmissions were defined according to the Swiss National Association for Quality Development in Hospitals and Clinics (ANQ) (10). It classifies as potentially preventable every unplanned readmission under 30 days of discharge for a pathology that was already known during the previous hospitalization.

Overall dissatisfaction was calculated as the sum of the item considered as unsatisfactory based on the Picker scheme. To determine a percentage value, this sum was divided by the total number of items and then multiplied by 100. A higher score indicated a greater level of dissatisfaction. This approach assumes that missing data implies an “absence of dissatisfaction.”

Construct validity was evaluated by considering established associations between patient satisfaction and demographic variables, hospital characteristics, and inpatient stay. Regarding demographic variables, age and male gender have been previously correlated with higher satisfaction (28–33). In terms of hospital characteristics, we made the assumption that departments with higher risk levels, such as intensive care and emergency care, might exhibit a negative association with satisfaction. Concerning inpatient stay variables, previous research has indicated negative association between satisfaction and factors such as LOS and patients complaints (34, 35).

### Statistical analyses

Descriptive statistics were counts and proportions for categorical variables and mean and standard deviation (SD) for continuous variables. Proportions of missing data were reported for every item across the three questionnaires.

To determine whether the data showed a similar number of components as the expected dimensions of the Picker patient experience questionnaire, we ran a Principal Component Analysis (PCA), using a screeplot and the Kaiser criterion to choose the number of components. Adequacy of PCA use was first assessed using the Kaiser–Meyer–Olkin and Bartlett tests.

To assess the adequacy and amount of information provided by each item, we used item response theory, with the information function for each value of the latent variable of patient satisfaction. Subsequently, the application of Rasch models, with a stringent threshold of 0.3, ensured a rigorous assessment of each item's fit within the overarching latent construct. The assessment of internal validity relied on Cronbach's alphas for each of the three questionnaires (adults, teenagers, children). Internal validity was computed on all questions, assuming an overall satisfaction latent variable. Construct validity was evaluated using univariable and multivariable linear regression, with patient satisfaction as the outcome. Both age and the count of negative and positive comments were treated as categorical variables to circumvent presuming linearity of their association. All analyses were performed using R version 4.2.2 (36).

## Results

### Cultural adaptation and pre-test

The forwards and backwards translation yielded minimal inconsistencies between translators and a general translation was agreed upon. The questionnaires were pretested on 34 adults, 23 parents, and 20 teenagers. During this pretest phase, feedback from numerous inpatients, particularly parents, suggested the addition of a question to evaluate the accessibility of care (including aspects such as accessibility via public transport, availability of parking facilities, both paid and free, etc.). Throughout the entire pre-test procedure, a sequence of five minor adjustments and updated questionnaire versions were introduced. For instance, feedback from parents of preschool-age children highlighted discomfort with a question inquiring about their child's participation in regular activities like sports or attending school, as their child was not yet attending. To address this, we incorporated a response option stating that the child was too young. All these modifications originated from suggestions provided by inpatients, aimed at refining the phrasing to ensure optimal item comprehensibility.

### Sample characteristics

*Adults:* Of the 97,488 patients discharged, 20,378 (20.9%) did not have a valid email or mobile phone number on record. Consequently, 77,110 individuals were reached out to, and from this group, 27,075 (response rate: 35.1%) provided responses to at least 7 questions. Within this subset, 25,626 (94.6%) opted to complete the questionnaire in French.

*Teenagers:* Of the 1,094 patients who were discharged, 65 (5.9%) lacked a valid email or mobile phone number. Thus, 1,029 were contacted, and among them, 311 (response rate: 30.2%) answered at least 7 questions. Out of these respondents, 293 (94.2%) selected the French version of the questionnaire.

*Children:* Among the 5,924 patients who were discharged, 436 (7.36 %) were without a valid email or mobile phone number. This led to 5,488 patients being contacted, and among this group, 1,782 (response rate: 32.5%) provided answers to at least 7 questions. Within this subgroup, 1,640 (92.0%) chose to complete the questionnaire in French.

There were more women among adults, and more boys among children, and an equal proportion of boys and girls among teenagers. Among adults, the median age was relatively youthful, at 52. Across all three patient populations, ~17% identified as allophones, signifying that French was not their primary language. The distribution of stays within specific departments was similar across all populations, except for emergency care, which was more frequent among teenagers (62.9%) and children (50.2%) compared to adults (29.1%) respondents. As anticipated, the proportion of patients admitted in psychiatry was low across all population groups (Table 1).

**Table 1:** Patients characteristics.

		**Adult**	**Teenagers**	**Children**
Overall		25,626	293	1,640
Gender (%)	F	15,132 (59.0)	149 (50.9)	701 (42.7)
	M	10,494 (41.0)	144 (49.1)	939 (57.3)
Age (median [IQR])		52.0 [35.0, 66.0]	14.0 [13.0, 15.0]	2.0 [0.0, 7.0]
Nationality (%)	CH	16,286 (64.2)	190 (65.5)	946 (58.9)
	Europe	6,833 (26.9)	66 (22.8)	479 (29.8)
	Other	2,260 (8.9)	34 (11.7)	182 (11.3)
Primary Language (%)	French	21,280 (83.0)	243 (82.9)	1,354 (82.6)
	Other	4,346 (17.0)	50 (17.1)	286 (17.4)
Positive comments	0	18,271 (71.3)	227 (77.5)	1,202 (73.3)
	1	6,326 (24.7)	55 (18.8)	361 (22.0)
	2/2+	903 (3.5)	11 (3.8)	77 (4.7)
	3+	126 (0.5)		
Negative comments	0	20,857 (81.4)	238 (81.2)	1,199 (73.1)
	1	3,610 (14.1)	45 (15.4)	307 (18.7)
	2/2+	857 (3.3)	10 (3.4)	98 (6.0)
	3+	302 (1.2)		36 (2.2)
Length of stay in days (median [IQR])		6.81 (17.89)	5.22 (6.20)	6.84 (18.68)
Preventable readmissions	Yes	1,242 (4.8)	16 (5.5)	75 (4.6)
Department of stay (%)^*^				
Surgery	Yes	9,477 (37.0)	90 (30.7)	397 (24.2)
Psychiatry	Yes	331 (1.3)	13 (4.4)	6 (0.4)
Emergency	Yes	7,460 (29.1)	147 (50.2)	1,031 (62.9)
Gynecology and Obstetrics	Yes	6,132 (23.9)		
Intensive care	Yes	312 (1.2)		
Pediatric medicine	Yes		79 (27.0)	984 (60.0)

^*^The sum of all percentages may exceed 100, since patients could stay in several departments during their complete sojourn. Percentage of stay is calculated per department in each population (Adults, Teens, Children).

### Questionnaires characteristics

Among adults, there was a notable level of dissatisfaction with continuity of care, as ~45% expressed not receiving information about (1) drug side effects, or (2) warnings and signals pertaining to their condition. Moreover, nearly 40% reported dissatisfaction with their level of engagement in treatment and care, as well as the coordination of care, which involved instances where healthcare professionals offered contradictory information. To a lesser extent (around 25% of dissatisfaction), respondents reported receiving inadequate emotional support across all three items within this dimension (Table 2).

**Table 2:** French version of the adults PPE-15 questionnaire: questions and loadings.

		**Adults n** = **25,626**			
**Dimension**	**Questions**	**Satisfaction**		**Missing %**	**Loadings**
		**Yes**	**No**		
Information and education	Doctors understandable	21,499 (84.2)	4,028 (15.8)	0.4	0.61
	Nurses understandable	20,867 (81.9)	4,618 (18.1)	0.6	0.62
Coordination of care	Contradictory information	16,550 (65.0)	8,897 (35.0)	0.7	0.42
Emotional support	Doctors address concerns	18,759 (73.6)	6,741 (26.4)	0.5	0.63
	Nurses address concerns	19,578 (76.9)	5,893 (23.1)	0.6	0.67
	Someone to discuss concerns	19,006 (74.7)	6,423 (25.3)	0.8	0.60
Respect of patient preferences	Doctors speaking as if you weren't there	21,925 (86.3)	3,482 (13.7)	0.9	0.32
	Implication in treatment and care	15,297 (60.6)	9,957 (39.4)	1.5	0.49
	Treated with dignity	22,229 (87.2)	3,263 (12.8)	0.5	0.59
Physical comfort	Presence of pain	17,894 (70.8)	7,382 (29.2)	1.4	
	Enough done to control pain	14,442 (81.1)	3,369 (18.9)	30.5^*^	0.55
Involvement of family and friends	Family opportunity to talk to doctors	20,041 (78.8)	5,398 (21.2)	0.7	0.46
	Family given information about condition and recovery	19,932 (78.6)	5,433 (21.4)	1.0	0.52
Continuity and transition	Purpose of drugs explained	21,021 (82.6)	4,421 (17.4)	0.7	0.51
	Told about side effects	14,556 (57.7)	10,680 (42.3)	1.5	0.45
	Told about danger signals	13,519 (53.5)	11,762 (46.5)	1.3	0.45
Overall impression		24,627 (96.7)	844 (3.3)	0.6	
Recommendation of hospital		19,649 (77.4)	5,732 (22.6)	1.0	
Overall score		71.9%	28.1%		
Cronbach's alpha		0.85			

^*^Percentage of missing responses is higher because this question was exclusively presented to patients who had indicated experiencing pain in response to the preceding question.

Results were relatively similar for teenagers, who were also frequently unsatisfied with continuity of care and the presentation of contradictory information by healthcare professionals. In addition, over 30% of teenagers expressed discontent with how doctors addressed their concerns. Parents also reported issues related to contradictory information, although they displayed a slightly lower level of dissatisfaction with continuity of care. However, over 40% of parents conveyed dissatisfaction with their involvement in the care of their children (Table 3).

**Table 3:** French version of the teenagers and children PPE-15 questionnaires: questions and loadings.

		**Teenagers n** = **293**				**Children n** = **1,640**			
**Dimension**	**Questions**	**Satisfaction (%)**		**Missing %**	**Loadings**	**Satisfaction (%)**		**Missing %**	**Loadings**
		**Yes**	**No**			**Yes**	**No**		
Information and education	Doctors understandable	251 (85.7)	42 (14.3)	0.0	0.46	1,463 (89.3)	175 (10.7)	0.1	0.42
	Nurses understandable	250 (85.6)	42 (14.4)	0.3	0.65	1,475 (90.3)	158 (9.7)	0.4	0.44
Coordination of care	Contradictory information	185 (63.4)	107 (36.6)	0.3	0.40	977 (60.1)	649 (39.9)	0.9	0.42
Emotional comfort	Doctors address concerns	200 (68.5)	92 (31.5)	0.3	0.72	1,131 (69.3)	500 (30.7)	0.5	0.60
	Nurses address concerns	224 (77.0)	67 (23.0)	0.7	0.22	1,244 (76.7)	377 (23.3)	1.2	0.20
Respect of patient preferences	Doctors speaking as if you were not there	244 (84.1)	46 (15.9)	1.0	0.16	1,398 (85.7)	234 (14.3)	0.5	0.24
	Treated with dignity	252 (86.3)	40 (13.7)	0.3	0.65	1,463 (89.4)	173 (10.6)	0.2	0.60
Physical comfort	Presence of pain	167 (74.9)	56 (25.1)	23.9	0.64	756 (75.1)	251 (24.9)	38.6	0.60
	Enough done to control pain	250 (85.9)	41 (14.1)	0.7	0.63	1,360 (83.3)	272 (16.7)	0.5	0.58
Continuity and transition	Purpose of drugs explained	246 (84.2)	46 (15.8)	0.3	0.42	1,459 (89.2)	176 (10.8)	0.3	0.46
	Told about side effects	161 (55.7)	128 (4.3)	1.4	0.41	954 (58.6)	674 (41.4)	0.7	0.35
	Told about danger signals	171 (59.0)	119 (41.0)	1.0	0.54	1,165 (71.3)	470 (28.7)	0.3	0.45
	Told about physical activity	200 (68.7)	91 (31.3)	0.7	0.46	1,302 (79.7)	332 (20.3)	0.4	0.34
Involvement of family and friends	Family given information about condition and recovery	227 (77.7)	65 (22.3)	0.3	0.61	1,357 (82.9)	279 (17.1)	0.2	0.61
Involvement of parents	Involved in care	Not a question in the teenager questionnaire				941 (57.9)	684 (42.1)	0.9	0.41
	Felt listened to	Not a question in the teenager questionnaire				1,297 (79.3)	338 (20.7)	0.3	0.69
Overall impression		280 (95.9)	12 (4.1)	0.3		1,583 (97.1)	47 (2.9)	0.6	
Recommendation of hospital		222 (76.3)	69 (23.7)	0.7		1,248 (76.7)	380 (23.3)	0.7	
Overall score		72.2%	27.8%			78.6%	21.4%		
Cronbach's alpha		0.82				0.80			

Within the subset of patients who provided responses to the questionnaires, instances of missing answers were infrequent, with a notable exception for the question regarding information about drug side effects for both adults and teenagers (6.0% and 8.2% respectively, as indicated in Tables 2, 3). Interestingly, nearly all parents responded to this specific question, and their reported dissatisfaction level was relatively high.

### Principal component analysis and factor analysis

**Figure 1:**
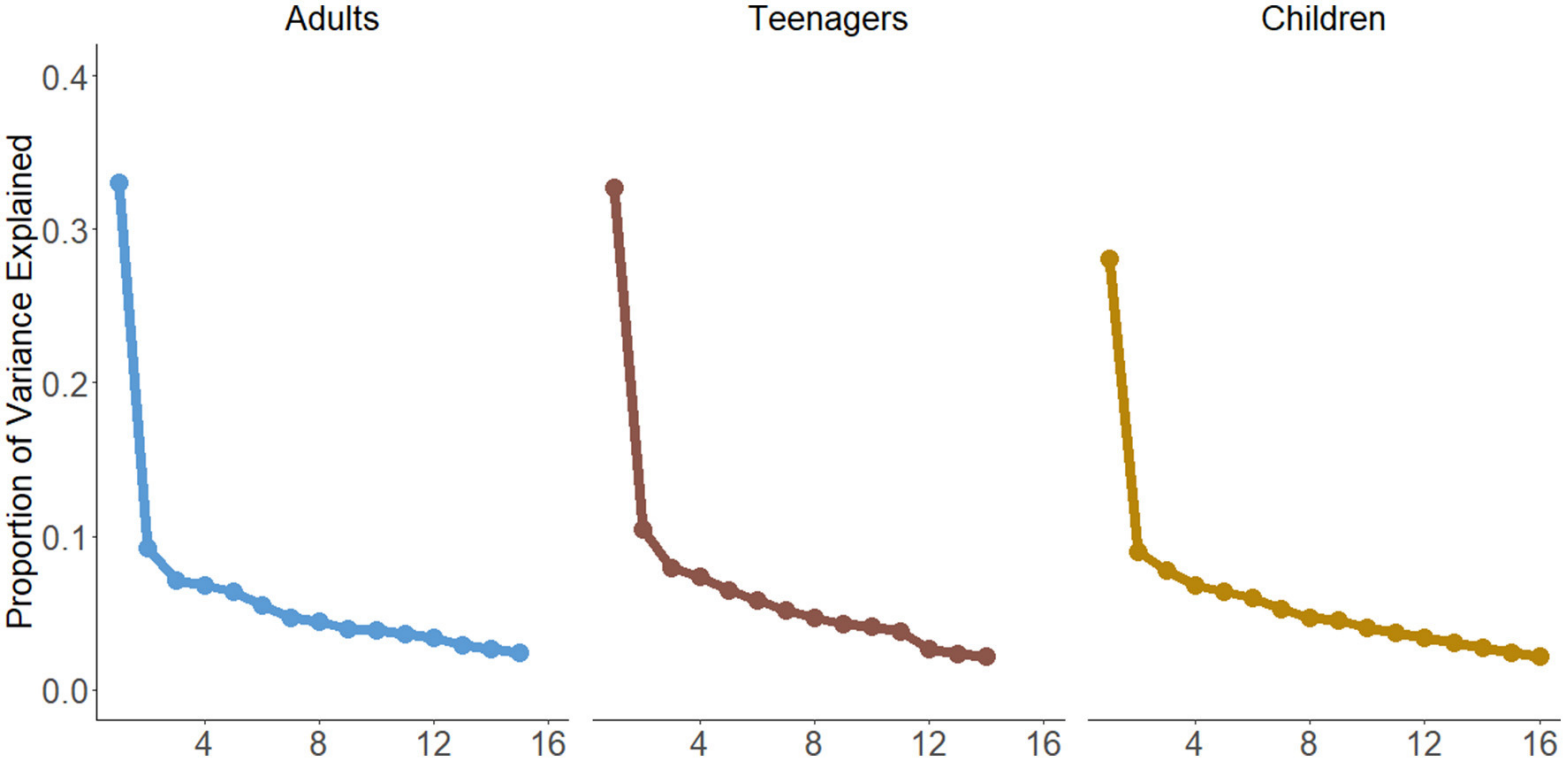
Principal component analysis for each population.

There were strong inter-items correlations in each population with Bartlett's tests always strongly significant (*p*s < 0.001) and Kaiser-Meyer-Olkin tests of 0.91 for adults, 0.87 for teenagers and 0.89 for children (Figure 1). Across each of the examined populations, PCA consistently yielded congruent outcomes, with a single prominent component evident. The loadings (Tables 2, 3), representing the correlation between the items and the underlying satisfaction construct, were predominantly satisfactory, often exceeding the threshold of 0.4. There were exceptions. The question regarding respect (“doctors speak in front of me as if I wasn't there”) exhibited poor performance across all categories. Furthermore, among teenagers and children, the item pertaining to emotional support (“nurses addressing concerns”) had low loading. Among parents of children, two continuity and transition-related items, “being told about side effects” and “being told about physical activity,” also displayed low association with the overall scale. Cronbach's alphas were 0.85 for adults, 0.82 for teenagers, and 0.80 for children.

### Rasch models

**Figure 2:**
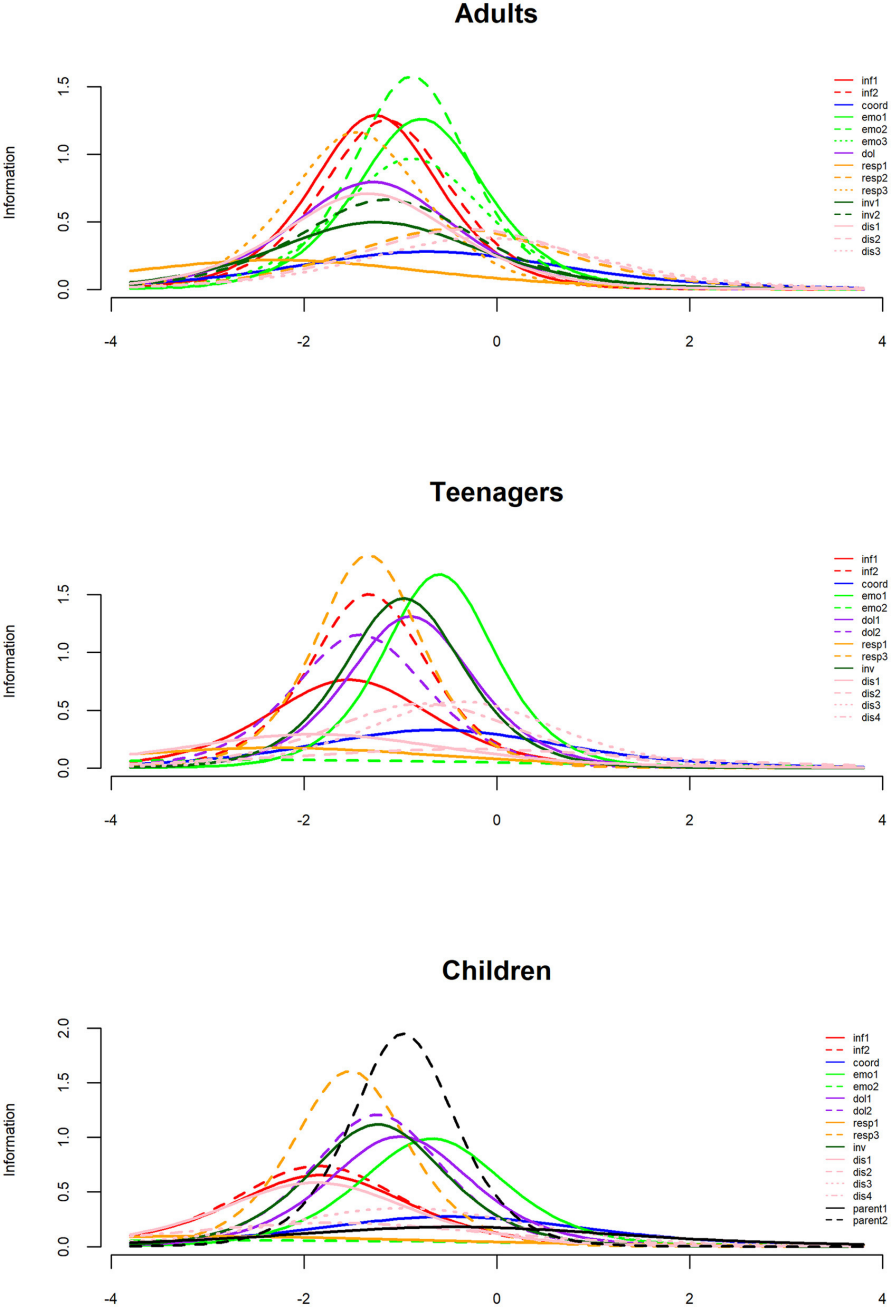
Information index for each population. Items are identified by their dimension: Inf = Information and education (2 items), coord, Coordination of care; emo, Emotional support; dol, Physical comfort; resp, Respect of patient preferences; inv, Involvement of family and friends; dis, Continuity and transitions; parent, Involvement of parents.

Most items had good information content and covered together a large range of dissatisfaction. Nevertheless, a few items demonstrated low information content. The question on coordination of care (“Sometimes in a hospital, one doctor or nurse will say one thing, and another will say something quite different. Did this happen to you?”) had low information for all three populations. Questions such as “Did you feel that friends and family were welcome to visit your child?” (emo2 on Figure 2) and “Did doctors talk to other hospital staff in front of you and your child, as if you were not there?” (resp1 on Figure 2) yielded minimal information for children and teenagers. This corroborated the findings obtained from the factor analysis.

**Figure 3:**
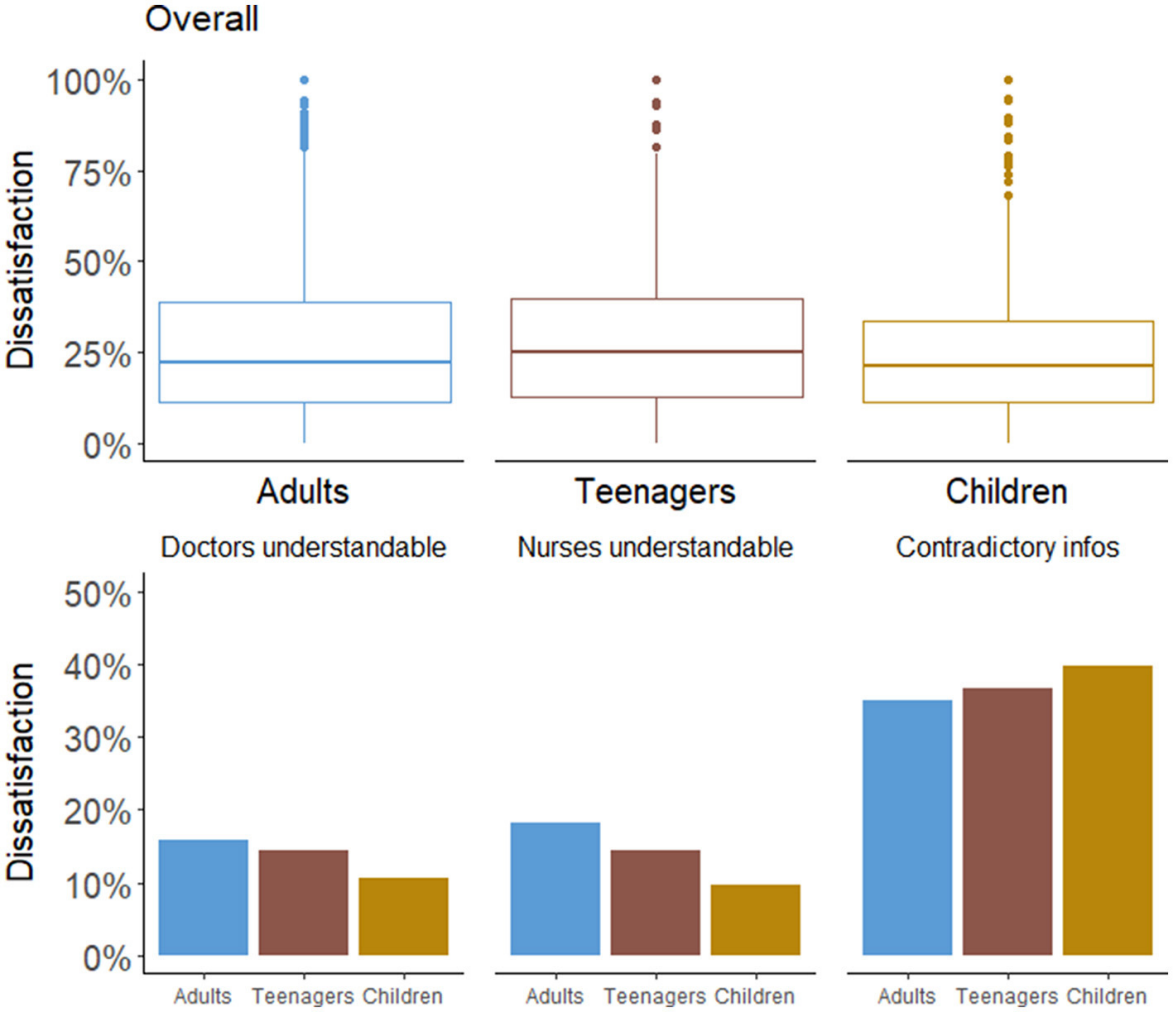
Distribution of dissatisfaction among populations overall (top panel) and for the specific three items (bottom panel).

While the overall level of satisfaction remained relatively similar across the three population groups, notable variability emerged within populations. This observation is exemplified by the responses to questions such as “When you had important questions to ask a doctor, did you get answers that you could understand?” and “When you had important questions to ask a nurse, did you get answers that you could understand?” Similarly, variation was also evident between questions, particularly in the context of the “Sometimes in a hospital, one doctor or nurse will say one thing, and another will say something quite different. Did this happen to you?” question, as indicated in Figure 3.

Among adults, dissatisfaction was more pronounced in terms of the clarity of responses received from nurses or doctors, in contrast to parents of children. Teenagers demonstrated a higher level of satisfaction than adults, though lower than parents. Conversely, adults expressed a slightly higher satisfaction level in relation to care coordination, with teenagers reporting lower satisfaction, and parents even lower levels.

### Construct validity

Among adults, dissatisfaction was higher among older patients (>80 years old), and patients with longer length of stay or preventable readmission. As could be expected, patients who wrote negative comments also rated satisfaction more negatively. These associations remained similar in the adjusted analysis, except for age, with patients over 40 years old reporting more satisfaction than younger adult patients, though this difference was smaller for patients 80 years or older.

Regarding specific departments, patients who stayed in the emergency care, intensive care, psychiatry, and rehabilitation departments reported higher levels of dissatisfaction, whereas patients who stayed in the gynecology and obstetrics department indicated lower levels of dissatisfaction.

Raw and adjusted analyses in both teenagers and parents of children showed a similar pattern of results than for adults, with negative comments, length of stay and preventable readmissions associated with higher dissatisfaction. The main difference related to the departments, with dissatisfaction lower in surgery and higher in general pediatrics. Passage in emergency care was associated with higher dissatisfaction among teenagers, but not among children. A difference specific to children was the strength of the association between preventable readmission and dissatisfaction, which was much lower and even became non-significant in the adjusted analysis (Table 4).

**Table 4:** Univariable and multivariable analysis association with dissatisfaction.

	**Univariable estimate [95% CI]**	**P-value**	**Multivariable estimate [95% CI]**	**P-value**
**Adults**				
Male gender	0.74^**^ [0.21, 1.27]	0.007	−0.89^**^ [−1.44, −0.34]	0.002
Age 40–60	0.01 [−0.64, 0.66]	0.97	−3.56^***^ [−4.23, −2.89]	< 0.001
Age 60–80	−0.42 [−1.09, 0.24]	0.21	−4.29^***^ [−5.01, −3.58]	< 0.001
Age >80	4.20^***^ [2.99, 5.42]	< 0.001	−1.90^**^ [−3.14, −0.67]	0.003
Length of stay	0.10^***^ [0.08, 0.11]	< 0.001	0.05^***^ [0.04, 0.06]	< 0.001
1 negative comment	12.63^***^ [11.90, 13.35]	< 0.001	13.24^***^ [12.54, 13.93]	< 0.001
2 negative comments	22.59^***^ [21.19, 23.98]	< 0.001	23.69^***^ [22.35, 25.03]	< 0.001
3+ negative comments	31.60^***^ [29.27, 33.92]	< 0.001	32.97^***^ [30.74, 35.19]	< 0.001
1 positive comment	−7.32^***^ [−7.93, −6.72]	< 0.001	−8.79^***^ [−9.35, −8.23]	< 0.001
2 positive comments	−9.56^***^ [−10.98, −8.15]	< 0.001	−10.30^***^ [−11.61, −9.00]	< 0.001
3+ positive comments	−9.72^***^ [−13.43, −6.01]	< 0.001	−11.55^***^ [−14.97, −8.13]	< 0.001
Preventable readmissions	5.81^***^ [4.58, 7.03]	< 0.001	4.01^***^ [2.89, 5.14]	< 0.001
**Stay in**				
Surgery	−0.55^*^ [−1.10, −0.01]	0.047	−0.42 [−1.02, 0.18]	0.17
Gynecology or obstetrics	−5.83^***^ [−6.44, −5.22]	< 0.001	−4.85^***^ [−5.71, −3.99]	< 0.001
Psychiatry	20.69^***^ [18.38, 23.01]	< 0.001	17.97^***^ [15.78, 20.15]	< 0.001
Rehabilitation	8.49^***^ [7.48, 9.49]	< 0.001	6.87^***^ [5.84, 7.90]	< 0.001
Intensive medicine	9.34^***^ [6.95, 11.73]	< 0.001	6.06^***^ [3.77, 8.35]	< 0.001
Emergency care	4.99^***^ [4.41, 5.56]	< 0.001	4.52^***^ [3.93, 5.10]	< 0.001
**Teenagers**				
Male gender	−5.18^*^ [−9.90, −0.46]	0.03	−3.53 [−8.20, 1.14]	0.14
Age 14	1.42 [−4.33, 7.18]	0.63	1.52 [−3.93, 6.97]	0.58
Age 15	5.37 [−0.55, 11.28]	0.08	3.88 [−1.71, 9.47]	0.17
Length of stay	0.69^***^ [0.31, 1.07]	< 0.001	0.50^*^ [0.11, 0.90]	0.01
Preventable readmissions	13.11^*^ [2.75, 23.47]	0.01	10.39^*^ [0.48, 20.30]	0.04
1 negative comment	5.42 [−1.02, 11.86]	0.10	4.99 [−1.23, 11.20]	0.12
2+ negative comments	25.66^***^ [12.87, 38.45]	< 0.001	27.95^***^ [15.28, 40.62]	< 0.001
1 positive comment	−4.78 [−10.88, 1.32]	0.13	−5.65 [−11.59, 0.29]	0.06
2+ positive comments	−5.64 [−18.17, 6.89]	0.38	−7.01 [−19.25, 5.23]	0.26
**Stay in**				
Surgery	−8.21^**^ [−13.28, −3.14]	0.002	−4.56 [−10.04, 0.93]	0.10
General pediatrics	8.50^**^ [3.23, 13.77]	0.002	3.84 [−2.36, 10.04]	0.23
Psychiatry	8.30 [−3.21, 19.81]	0.16	1.65 [−10.30, 13.61]	0.79
Emergency care	1.31 [−3.45, 6.06]	0.59	−1.66 [−6.64, 3.33]	0.52
**Children**				
Male gender	0.55 [−1.24, 2.33]	0.55	1.02 [−0.62, 2.67]	0.22
Age 3–5	0.88 [−1.60, 3.37]	0.49	3.40^**^ [1.10, 5.70]	0.004
Age 6–8	−3.76^**^ [−6.46, −1.06]	0.006	−0.64 [−3.19, 1.91]	0.62
Age 9–12	−2.04 [−4.49, 0.42]	0.10	1.37 [−0.99, 3.73]	0.26
Length of stay	0.09^***^ [0.04, 0.13]	< 0.001	0.09^***^ [0.04, 0.13]	< 0.001
Preventable readmissions	4.48^*^ [0.25, 8.71]	0.04	3.24 [−0.68, 7.17]	0.11
1 negative comment	9.73^***^ [7.57, 11.89]	< 0.001	11.03^***^ [8.90, 13.17]	< 0.001
2 negative comments	16.12^***^ [12.58, 19.67]	< 0.001	18.02^***^ [14.53, 21.52]	< 0.001
3+ negative comments	27.09^***^ [21.38, 32.80]	< 0.001	28.88^***^ [23.31, 34.46]	< 0.001
1 positive comment	−4.18^***^ [−6.32, −2.05]	< 0.001	−6.82^***^ [−8.84, −4.80]	< 0.001
2+ positive comments	−7.66^***^ [−11.84, −3.47]	< 0.001	−9.69^***^ [−13.57, −5.81]	< 0.001
**Stay in**				
Surgery	−4.75^***^ [−6.80, −2.69]	< 0.001	−0.83 [−3.10, 1.43]	0.47
General pediatrics	6.04^***^ [4.25, 7.82]	< 0.001	3.94^***^ [1.82, 6.05]	< 0.001
Emergency care	1.29 [−0.54, 3.12]	0.17	0.16 [−1.65, 1.96]	0.87

^*^P < 0.05, ^**^P < 0.005,^***^P < 0.001.

## Discussion

This validation study encompassed the translation and adapation of the PPE-15 satisfaction scale for three distinct French-speaking populations : adults, teenagers, and children. The scales exhibited good internal consistency for an overall dissatisfaction score with Cronbach's alpha values ≥ 0.8 across all three scales. The PCA yielded a single latent variable for each scale. Thus, the original seven dimensions were not observed in the set of 15 questions. While a validation study involving a Spanish version of the PPE-15 reported four components to satisfaction (24) their loadings displayed a primary component incorporating most items, along with three components with minimal loadings surpassing their selected threshold (0.32). Collectively, these findings support the existence of a single general latent satisfaction variable present across all three populations and underscore the potential limitation of the 15 items to accurately assess specific dimensions of satisfaction. Though this implies that the PPE-15 does not measure reliably each satisfaction dimension, individual items could still be used, but at the risk of having imprecise measures of specific dimension.

Item performance discrepancies were evident among each population group. In the adult population, all items were relevant, except the item concerning patient respect (“Doctors speaking as if I wasn't there”), which exhibited low informativeness and a weak loading. This item also demonstrated limited reliability for both pediatric populations. This finding replicates the original PPE-15 validation article findings (21), where the item displayed a relatively high loading in Germany and Switzerland but failed to meet the threshold in Sweden and the USA. In that original study, the item was retained in the final questionnaire due to its strong face validity. Nevertheless, this recurring finding suggests the potential necessity for reformulation. This might be attributed to the fact that patients may not consistently view doctors speaking as if they were not there as unsatisfactory, across cultures and contexts. This question was initially designed to assess respect. Considering the observed performance issues, we propose rephrasing the question to incorporate a negative aspect that could potentially better reflect patient dissatisfaction. For instance, the question could be reformulated as follows: “Did you feel doctors were ignoring you while speaking in front of you as if you weren't there?” This modification aims to enhance the question's sensitivity to patient dissatisfaction with the specific situation. Instances arise where questions possess high face validity a priori, yet their formulation results in low reliability. For instance, the question addressing one's involvement in their own care was modified to emphasize dissatisfaction with the extent of involvement. Despite this, we opted to retain this item as is, aiming to maintain consistency and comparability with the other PPE-15 versions.

In both pediatric populations, an additional item performed poorly (Emotion support – Nurses addressing concerns), underlining the importance of a validation in specific populations. The variation in item functioning for different age groups is expected, as their care-related expectations differ substantially (37, 38). Teenagers and parents of young children may be more accepting of the nurses not addressing the concerns of the pediatric patient because they talk to the parents, allowing the latter to provide support. Rasch models showed that several questions delivered minimal information to an overall satisfaction construct, particularly in the case of children and teenagers. Despite these questions' limited utility for the overall satisfaction measure, they were retained to preserve questionnaire integrity and consistency with existing literature, but also to maintain content validity by assessing all dimensions of satisfaction. Our findings open the way to produce an even shorter satisfaction questionnaire, aimed at measuring only the overall satisfaction, and not specific dimensions.

During the pretest phase, patients and parents suggested that certain essential topics influencing their hospitalization satisfaction were missing. For example, the accessibility of hospital infrastructure supporting ease of access for their family was raised in both children and teenager situations. Concrete examples of questions regarding parking accessibility were suggested. While these questions are relevant, they don't assess experience with care and thus were not included in the scale.

Overall, associations with other constructs were aligned with anticipated relationships. Factors most consistently associated to dissatisfaction included LOS, preventable readmissions, and the presence of negative and positive comments. The positive association with LOS supports the idea that extended hospital stays increase the likelihood of negative events at some point. The commonly observed age-related association with dissatisfaction was similarly identified in adults as well as a strong association between preventable readmissions and dissatisfaction, reinforcing the construct validity of the adapted questionnaire. Gender associations with dissatisfaction appeared inconsistent, with differences between univariable and multivariable analyses in adults and conflicting associations when comparing children, teenagers, and adults. This finding parallels those reported in literature reviews on factors associated with patient satisfaction (16, 32) and highlight the complexity of gender associations, particularly within pediatric population where the gender of the child may differ from the gender of the person completing the questionnaire.

### Strengths and limitations

The main strength of this study is the substantial patient sample and the concurrent measurement of satisfaction in three distinct populations. Indeed, residents and nurses rotate across divisions and departments. Thus, a concurrent evaluation for all populations in the same hospital provides stronger evidence for a similar or different item functioning across populations. The substantial sample size provides greater confidence in the good construct validity, mirroring the established correlations between satisfaction and independent variables. The main limitation lies in the use of data from a single hospital, potentially constraining the generalizability of our findings, especially to other French-speaking region in Europe, North America, or Africa. Further studies should be conducted in other French-speaking centers to ensure the questionnaire's cross-cultural validity prior to implementing in different settings. A second limitation is the lack of test-retest reliability due to the absence of longitudinal data. While the adult version of the PPE-15 showed good test-retest reliability, with an ICC of 0.9, further investigation should be conducted to attempt to replicate this finding with the French culturally adapted PPE-15 in all three populations (39). Another limitation is selection bias due to the nature of the data collection. Contact by email or text message, the electronic response format, and a relatively low response rate may result in the selection of a different population compared to the overall patient population. Response rates are often lower among men and individuals with low socio-economic status (40, 41). To mitigate the potential effect of this selection bias on test clarity, particular attention was paid to including patients from various socioeconomic statuses in the pretest phase.

## Conclusion

The French adaptations of the PPE-15 scales for adults, teenagers and parents of children inpatients have proven to be valid and reliable for evaluating the general level of satisfaction among hospitalized individuals regarding their stay after discharge. Nonetheless, some questions might benefit from rephrasing to enhance their reliability. Implementing this tool in other French-speaking hospitals would contribute to a more comprehensive understanding of the effects of quality improvement programs and facilitate benchmarking.

## Data availability statement

The data analyzed in this study is subject to the following licenses/restrictions: Due to patient data confidentiality the datasets analyzed aren't available. The French culturally adapted questionnaires are in the Supplementary material. Requests to access these datasets should be directed to clement.buclin@hcuge.ch.

## Ethics statement

Ethical approval was not required for the study involving humans in accordance with the local legislation and institutional requirements. Written informed consent to participate in this study was not required from the participants or the participants' legal guardians/next of kin in accordance with the national legislation and the institutional requirements.

## Author contributions

CB: Writing—original draft, Writing—review & editing. AU: Writing—review & editing. JD: Writing—review & editing. AI: Writing—review & editing. JS: Writing—review & editing. GH: Writing—review & editing. SC: Conceptualization, Writing—review & editing. DC: Conceptualization, Writing—original draft, Writing—review & editing.
