# Supplementary material for: Validation of French versions of the 15-item picker patient experience questionnaire for adults, teenagers, and children inpatients
Source: Front Public Health. 2024 Feb 19;12:1297769. doi: 10.3389/fpubh.2024.1297769 (PMC10910618; doi:10.3389/fpubh.2024.1297769)
Supplement: Supplementary file 1 [file Table_1.pdf]

Supplementary file 1: French PPE-15 : Adults

Answers that are to be classified as dissatisfactory are in **bold**

1. [Information and education 1] Lorsque vous avez posé des questions importantes à un.e médecin, avez-vous obtenu des réponses que vous pouviez comprendre ?

Oui, toujours

Oui, parfois

Non

Je n'ai pas eu besoin de poser de questions

2. [Information and education 2] Lorsque vous avez posé des questions importantes à un.e infirmier.e, avez-vous obtenu des réponses que vous pouviez comprendre ?

Oui, toujours

Oui, parfois

Non

Je n'ai pas eu besoin de poser de questions

3. [Coordination of care] Dans un hôpital, il arrive qu'un.e médecin ou un.e infirmier.e dit une chose et un.e autre dit quelque chose de tout à fait différent. Cela vous est-il arrivé ?

Oui, souvent

Oui, parfois

Non

4. [Emotion comfort 1] Si vous aviez des inquiétudes ou des craintes au sujet de votre état ou de vos traitements, un.e médecin en a-t-il parlé avec vous ?

Oui, complètement

Oui, dans une certaine mesure

Non

Je n'avais ni inquiétude ni crainte

5. [Emotion comfort 2] Si vous aviez des inquiétudes ou des craintes au sujet de votre état ou de vos traitements, un.e infirmier.e en a-t-il parlé avec vous ?

Oui, complètement

Oui, dans une certaine mesure

Non

Je n'avais ni inquiétude ni crainte

6. [Respect of patient preferences 1] Les médecins ont-ils parlé devant vous comme si vous n'étiez pas là ?

Oui, souvent

Oui, parfois

Non

7. [Respect of patient preferences 2] Auriez-vous voulu être plus impliqué.e dans les décisions concernant vos soins et vos traitements ?

Oui, absolument

Oui, dans une certaine mesure

Non

8. [Respect of patient preferences 3] Dans l'ensemble, vous êtes-vous senti.e traité.e avec respect et dignité pendant votre séjour à l'hôpital ?

Oui, toujours

Oui, parfois

Non

9. [Emotion comfort 3] Avez-vous trouvé quelqu'un parmi le personnel de l'hôpital à qui parler de vos préoccupations ?

Oui, absolument

Oui, dans une certaine mesure

Non

Je n'avais aucune préoccupation

10. [Physical comfort 1] Vous est-il arrivé d'avoir des douleurs?

Oui

Non

11. [Physical comfort 2] Pensez-vous que le personnel de l'hôpital a fait tout son possible pour aider à contrôler votre douleur ?

Oui, absolument

Oui, dans une certaine mesure

Non

12. [Involvement of family and friends] Si votre famille ou un proche souhaitait parler à un.e médecin, ont-ils eu suffisamment l'opportunité de le faire ?

Oui, absolument

Oui, dans une certaine mesure

Non

Aucun membre de ma famille ou proche n'était impliqué

Aucun membre de ma famille ou proche ne voulait ou n'avait besoin d'information

Je ne voulais pas que ma famille ou mes proches parlent à un médecin

13. [Involvement of family and friends 2] Les médecins ou les infirmièr.e.s ont-ils donné à votre famille ou à un proche toute l'information dont ils avaient besoin pour vous aider à vous rétablir ?

Oui, absolument

Oui, dans une certaine mesure

Non

Aucun membre de ma famille ou proche n'était impliqué

Aucun membre de ma famille ou proche ne voulait ou n'avait besoin d'information

14. [Continuity and transition 1] Un membre du personnel vous a-t-il expliqué l'utilité des médicaments à prendre à la maison d'une manière que vous pouviez comprendre ?

Oui, complètement

Oui, dans une certaine mesure

Non

Je n'avais pas besoin d'explication

Je n'avais pas de médicaments

15. [Continuity and transition 2] Un membre du personnel vous a-t-il parlé des effets secondaires des médicaments à surveiller une fois rentré chez vous ?

Oui, complètement

Oui, dans une certaine mesure

Non

Je n'avais pas besoin d'explication

16. [Continuity and transition 3] Quelqu'un vous a-t-il parlé des signaux d'alerte (symptômes qui nécessitent de contacter un professionnel de santé) à surveiller une fois chez vous ?

Oui, complètement

Oui, dans une certaine mesure

Non

Supplementary file 2: French PPE-15 : Teenager (Children > 12 French)

1. [Information and education 1] Est-ce que les médecins t'ont parlé de comment ils allaient s'occuper de toi d'une manière que tu pouvais comprendre ?

Oui, toujours

Oui, parfois

Non

Je n'ai pas eu besoin de poser de questions

2. [Information and education 2] Est-ce que les infirmier.e.s t'ont parlé de comment ils allaient s'occuper de toi d'une manière que tu pouvais comprendre ?

Oui, toujours

Oui, parfois

Non

Je n'ai pas eu besoin de poser de questions

3. [Coordination of care] Dans un hôpital, il arrive qu'un.e médecin ou un.e infirmier.e dit une chose et un.e autre dit quelque chose de tout à fait différent. Cela t'est-il arrivé ?

Oui, souvent

Oui, parfois

Non

4. [Emotion comfort 1] Penses-tu que le personnel de l'hôpital a tout fait pour diminuer tes peurs et tes craintes pendant ton séjour à l'hôpital ?

Oui, complètement

Oui, dans une certaine mesure

Non

Je n'avais ni inquiétude ni crainte

5. [Respect of patient preferences 1] Les médecins ont-ils parlé devant toi comme si tu n'étais pas là ?

Oui, souvent

Oui, parfois

Non

6. [Respect of patient preferences 2] Dans l'ensemble, t'es tu senti.e traité.e avec respect et dignité pendant ton séjour à l'hôpital ?

Oui, toujours

Oui, parfois

Non

7. [Physical comfort 1] T'es-t-il arrivé d'avoir des douleurs?

Oui

Non

8. [Physical comfort 2] Penses-tu que le personnel de l'hôpital a fait tout son possible pour aider à contrôler ta douleur ?

Oui, absolument

Oui, dans une certaine mesure

Non

9. [Involvement of family and friends] Les médecins ou les infirmièr.e.s ont-ils donné à ta famille ou à un proche toute l'information dont ils avaient besoin pour t'aider à te rétablir ?

Oui, absolument

Oui, dans une certaine mesure

Non

Aucun membre de ma famille ou proche n'était impliqué

Aucun membre de ma famille ou proche ne voulait ou n'avait besoin d'information

10. [Continuity and transition 1] Un membre du personnel t'a-t-il expliqué l'utilité des médicaments à prendre à la maison d'une manière que tu pouvais comprendre ?

Oui, complètement

Oui, dans une certaine mesure

Non

Je n'avais pas besoin d'explication

Je n'avais pas de médicaments

11. [Continuity and transition 2] Un membre du personnel t'a-t-il parlé des effets secondaires des médicaments à surveiller une fois rentré chez toi ?

Oui, complètement

Oui, dans une certaine mesure

Non

Je n'avais pas besoin d'explication

12. [Continuity and transition 3] Quelqu'un t'a-t-il parlé des signaux d'alerte à surveiller une fois chez toi ? (symptômes qui nécessitent de contacter un professionnel de santé)

Oui, complètement

Oui, dans une certaine mesure

Non

13. [Involvement of family and friends] Est-ce que tu as eu l'impression que tes amis et ta famille étaient les bienvenus pour te rendre visite ?

Oui, absolument

Oui, dans une certaine mesure

Non

Non applicable (COVID 19, maladies contagieuses)

14. [Continuity and transition 4] Quelqu'un t'a-t-il indiqué quand tu pouvais reprendre tes activités habituelles, comme faire du sport ou retourner à l'école ?

Oui, complètement

Oui, dans une certaine mesure

Non

15. [Emotion comfort 2] Pendant ton séjour à l'hôpital, le personnel infirmier t'a-t-il aidé quand tu en avais besoin ?

Oui, absolument

Oui, dans une certaine mesure

Non

Supplementary file 3: French PPE-15 : Children ≤ 12 French

1. [Information and education 1] Est-ce que les médecins ont parlé à votre enfant de la manière dont ils allaient s'occuper de lui dans un langage compréhensible pour lui ?

Oui, toujours

Oui, parfois

Non

Mon enfant est trop jeune

2. [Information and education 2] Est-ce que les infirmier.e.s ont parlé à votre enfant de la manière dont ils.elles allaient s'occuper de lui dans un langage compréhensible pour lui ?

Oui, toujours

Oui, parfois

Non

Mon enfant est trop jeune

3. [Coordination of care] Dans un hôpital, il arrive qu'un.e médecin ou un.e infirmier.e dit une chose et un.e autre dit quelque chose de tout à fait différent. Cela vous est-il arrivé ?

Oui, souvent

Oui, parfois

Non

4. [Emotion comfort 1] Pensez-vous que le personnel de l'hôpital a tout fait pour apaiser les peurs et les craintes de votre enfant pendant son séjour à l'hôpital ?

Oui, complètement

Oui, dans une certaine mesure

Non

Mon enfant n'avait ni inquiétude ni crainte

5. [Respect of patient preferences 1] Les médecins ont-ils parlé devant vous et votre enfant comme si vous n'étiez pas là ?

Oui, souvent

Oui, parfois

Non

6. [Respect of patient preferences 2] Dans l'ensemble, avez-vous eu l'impression que votre enfant a été traité avec respect et dignité pendant son séjour à l'hôpital ?

Oui, toujours

Oui, parfois

Non

7. [Physical comfort 1] Votre enfant a-t-il eu des douleurs?

Oui

Non

8. [Physical comfort 2] Pensez-vous que le personnel de l'hôpital a fait tout son possible pour aider à contrôler la douleur de votre enfant ?

Oui, absolument

Oui, dans une certaine mesure

Non

9. [Involvement of family and friends] Les médecins ou les infirmier.e.s vous ont-ils donné toute l'information dont vous aviez besoin pour aider votre enfant à se rétablir ?

Oui, absolument

Oui, dans une certaine mesure

Non

10. [Continuity and transition 1] Un membre du personnel vous a-t-il expliqué l'utilité des médicaments à prendre à la maison d'une manière que vous pouviez comprendre ?

Oui, complètement

Oui, dans une certaine mesure

Non

Nous n'avions pas besoin d'explication

Mon enfant n'avait pas de médicaments

11. [Continuity and transition 2] Un membre du personnel vous a-t-il parlé des effets secondaires des médicaments à surveiller une fois rentré chez vous ?

Oui, complètement

Oui, dans une certaine mesure

Non

Nous n'avions pas besoin d'explication

12. [Continuity and transition 3] Un membre du personnel vous a-t-il parlé des signaux d'alerte (symptômes qui nécessitent de contacter un professionnel de santé) à surveiller une fois chez vous ?

Oui, complètement

Oui, dans une certaine mesure

Non

13. [Emotion comfort 2] Avez-vous eu le sentiment que les amis et la famille étaient les bienvenus pour rendre visite à votre enfant ?

Oui, toujours

Oui, dans une certaine mesure

Non

Non applicable (COVID 19, maladies contagieuses)

14. [Continuity and transition 4] Quelqu'un vous a-t-il indiqué quand votre enfant pouvait reprendre ses activités habituelles, comme faire du sport ou retourner à l'école ?

Oui, absolument

Oui, dans une certaine mesure

Non

Mon enfant est trop jeune

15. [Physical comfort 3] Durant le séjour de votre enfant à l'hôpital, les infirmières ont-elles apporté à votre enfant l'aide dont il avait besoin ?

Oui, absolument

Oui, dans une certaine mesure

Non

16. [Parent involvement 1] Auriez-vous voulu être plus impliqué.e dans les décisions concernant les soins et les traitements de votre enfant ?

Oui, absolument

Oui, dans une certaine mesure

Non

17. [Parent involvement 2] Pensez-vous que le personnel qui s'occupait de votre enfant était à votre écoute?

Oui, toujours

Oui, parfois

Non
